# Trajectory Patterns of Three Lifestyle Behaviors and Subsequent Health Conditions in Japanese Adults: A Retrospective Longitudinal Study Using a Health Checkup Database

**DOI:** 10.31662/jmaj.2024-0076

**Published:** 2024-10-03

**Authors:** Tetsuya Tajima, Rieko Kanehara, Makoto Fujii, Shiori Tanaka, Jun Umezawa, Yuko Ohno, Manami Inoue

**Affiliations:** 1 Division of Cohort Research, National Cancer Center Institute for Cancer Control, Tokyo, Japan; 2 Division of Health Science, Graduate School of Medicine, Osaka University, Suita, Japan; 3 Division of Prevention, National Cancer Center Institute for Cancer Control, Tokyo, Japan

**Keywords:** lifestyle behavior, trajectory analysis, health condition, Japan

## Abstract

**Introduction:**

Although the associations between lifestyle behavioral changes over time and the risks of cancer and cardiovascular diseases are documented worldwide, evidence specific to the Japanese population remains limited. This study aimed to elucidate the trajectories of lifestyle behaviors and their associations with health conditions.

**Methods:**

We analyzed health checkup data from the Japan Medical Data Center Claims Database from 2005 to 2019, specifically those of individuals who underwent 10 consecutive annual checkups. We focused on smoking, frequency of drinking alcohol, and regular exercise habits as the exposure factors. A group-based trajectory model was employed to estimate the patterns of single and multiple trajectories for three exposures. Furthermore, a linear mixed-effects model was used to assess the association between trajectory patterns and longitudinal changes in health conditions (body mass index, systolic blood pressure [sBP], LDL-cholesterol, and HbA1c).

**Results:**

This study included 51,064 Japanese subjects aged 20-59 years at their initial health checkup. We identified seven trajectory groups (Groups 1-7) that represented a high percentage of subjects in the following order: Group 3 (inactive, 31.0%), Group 5 (long-term smoking, 26.9%), and Group 2 (daily drinkers, 11.0%). The only lifestyle behavioral change observed was smoking cessation. Groups 3 and 5 exhibited higher sBP (Group 3: β = 1.18, standard error [SE] = 0.60, p = 0.05; Group 5: β = 1.33, SE = 0.61, p < 0.05) and LDL-cholesterol levels (Group 3: β = 3.80, SE = 1.36, p < 0.05; Group 5: β = 3.04, SE = 1.37, p < 0.05) than the nonsmoking, nondrinking, and regular exercise groups. Group 2 exhibited significantly high sBP (β = 2.43, SE = 0.62, p < 0.001), with an observed interaction effect over time (p < 0.05).

**Conclusions:**

Regular exercise and abstinence from smoking and drinking may be essential to avoid deterioration of health conditions.

## Introduction

Lifestyle behaviors such as smoking, alcohol consumption, and physical inactivity are important risk factors for cancer and cardiovascular diseases in Japan, leading to increased mortality rates associated with these conditions ^(1), (2)^. To decrease the disease burden in Japan, it is essential to implement public health measures that encourage individuals to adopt a healthy lifestyle. Notably, the prevalence of lifestyle behaviors in Japan has changed over time, as indicated by the National Health and Nutrition Survey ^(3)^. This change may be influenced by health measures and the westernization of Asian diets ^(4)^.

Changes in lifestyle behaviors are associated with the risks of cancer and cardiovascular diseases. For example, a Korean cohort study reported that cancer incidence and mortality differed according to smoking behavior trajectories ^(5)^. A pooled analysis of Japanese cohort studies revealed that individuals who had quit smoking for a longer period had a comparable cancer risk to nonsmokers ^(6)^. In Denmark, research involving postmenopausal women demonstrated that those with increased alcohol consumption over a 5-year period had a higher risk of breast cancer and a lower risk of coronary heart disease than those with a stable alcohol intake ^(7)^. Moreover, cohort studies in Korea and China have reported that increased physical activity over time reduces the risk of cardiovascular diseases ^(8), (9)^. Hsu et al. used data from the Taiwan Longitudinal Study of the Elderly to examine the longitudinal trajectory patterns of smoking, alcohol consumption, physical activity, and periodic health checkups among individuals aged ≥60 years ^(10)^. Previous studies worldwide studies have reported the trajectories of lifestyle behaviors and their associations with the risk of cancer and cardiovascular disease.

However, studies on the association between changes in lifestyle behaviors over time and health conditions in the Japanese population are insufficient ^(11), (12)^. Two previous studies in Japan that investigated this association were limited to body mass index (BMI), diabetes, and older individuals. Therefore, there is a need to examine the trajectories of various lifestyle behaviors and health conditions among the Japanese population to develop further prevention measures for noncommunicable diseases.

We focused on three lifestyle behaviors, namely, smoking, alcohol consumption, and regular exercise, which are risk factors for cancer and cardiovascular diseases ^(1), (2)^. This study aimed to estimate the trajectory patterns of these lifestyle behaviors in Japanese adults and examine their association with health conditions, including BMI, systolic blood pressure (sBP), LDL-cholesterol, and HbA1c.

## Materials and Methods

### Study data source and subjects

This retrospective longitudinal study utilized health checkup data from the Japan Medical Data Center (JMDC) Claims Database provided by JMDC Inc. (Tokyo, Japan). The study period spanned from April 2005 to March 2019. The JMDC database collects claims (inpatient, outpatient, and prescription) and health checkup data from various health insurance societies in Japan ^(13), (14)^. The insurance societies that contribute this data include “Association/Union Administered Health Insurance,” which is one of the multiple health insurance systems in Japan. This insurance system mainly covers employees and their dependents in large-scale enterprises, such as those with ≥700 employees in a single establishment or ≥3,000 employees in multiple establishments.

**Figure 1.**
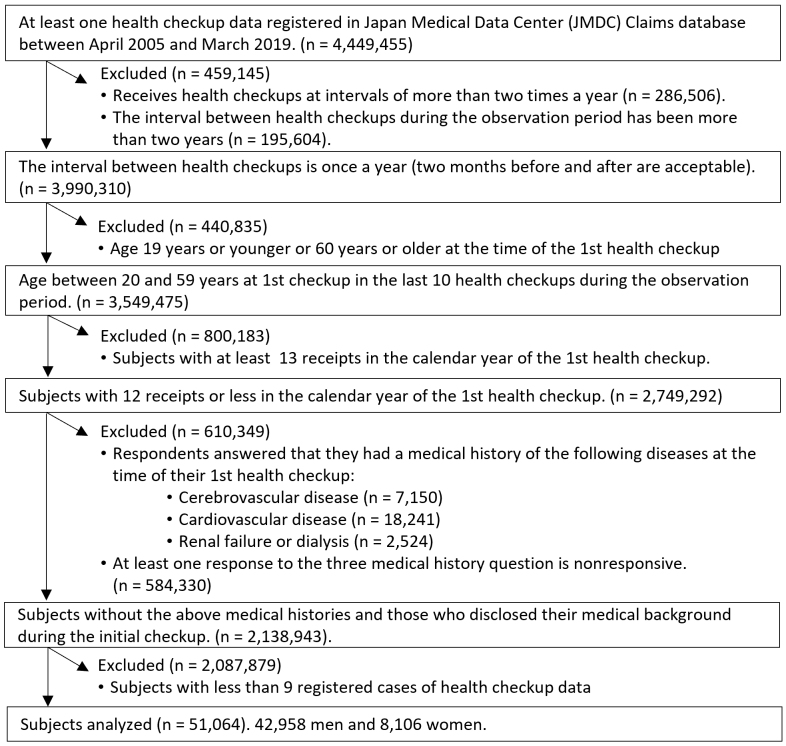
Flowchart of the study subjects.

The dataset used in this study consisted of 4,449,455 individuals, including 2,699,407 (61%) men and 1,750,048 women (39%) who underwent at least one health checkup during the observation period. However, certain exclusions were made to ensure data consistency and reliability. Employees in hazardous occupations who were required to undergo biannual health checkups were excluded due to their different characteristics (n = 286,506). Individuals who had at least one health checkup with an interval exceeding 2 years within the observation period were also excluded (n = 195,604). Furthermore, individuals whose age at their initial health checkup was ≤19 or ≥60 years were excluded (n = 440,835). Because of concerns about inconsistent data resulting from changes in health insurance societies related to retirement, we focused on the population below the age of 60. Notably, mandatory retirement typically occurs between the ages of 60 and 65 years in Japan in accordance with the Act on Stabilization of Employment of Elderly Persons. The reason for excluding individuals below the age of 19 was that smoking and alcohol consumption were prohibited by law for this age group in Japan. Individuals with more than 12 claims in the same calendar year as their initial health checkup were also excluded as it may have interfered with health behavior patterns during the study period (n = 800,183). This cutoff value represents the 75th percentile of claims across all health checkup recipients. In addition, those with existing medical histories related to cerebrovascular diseases, cardiovascular diseases, or renal failure/dialysis as well as those who did not disclose their medical background during the initial checkup were excluded (n = 610,349). Moreover, subjects with nine or fewer health checkups over the 14-year observation period were excluded to ensure sufficient reliability in the detection of trajectory patterns (n = 2,087,879). Among the excluded population, 2,479 subjects died during the observation period. For subjects with a total number of health checkups between 11 and 14, data from the last 10 health checkups were utilized. The final cohort for analysis comprised 51,064 individuals, including 42,958 (84%) men and 8,106 (15%) women (Figure 1). A sensitivity analysis, including subjects who had 1 year or more between health checkups, was also conducted (Supplementary Tables 5.1 and 5.2 and Supplementary Figures 5.1 and 5.2).

### Lifestyle behavior variables

This study focused on three lifestyle behaviors: smoking, alcohol consumption, and regular exercise. These behaviors were evaluated using a self-administered questionnaire during the annual health checkups. Smoking was defined as “current smoking” if the subject had smoked at least 100 cigarettes or had smoked for at least 6 months and smoked in the last month. The frequency of alcohol consumption was categorized into three groups: 1) every day, 2) occasionally, and 3) rarely. Regular exercise was defined as engaging in light sweat-inducing exercise for 30 min or more per session for at least 2 days per week for at least 1 year. Smoking and regular exercise were coded on a nominal scale (yes/no).

### Health condition variables

We evaluated health conditions including BMI (kg/m^2^), sBP (mmHg), LDL-cholesterol (mg/dL), and HbA1c (%). These variables were obtained from anthropometric and laboratory data collected during the health checkup and treated as continuous variables. HbA1c measurements were based on the National Glycohemoglobin Standardization Program.

### Statistical analysis

We used the group-based trajectory model (GBTM) with the Stata “traj” plugin to estimate the trajectory patterns of lifestyle behaviors ^(15), (16)^. This statistical approach aims to cluster the longitudinal data into groups with similar changing trajectories ^(17)^. Initially, we individually analyzed three lifestyle behaviors to estimate single-exposure trajectories. For smoking and regular exercise, which had binary outcomes, we used a logistic regression model. Conversely, we chose the Poisson model for alcohol consumption owing to its categorical nature. The optimal number of trajectory groups was determined by comparing Bayesian information criterion values, ensuring that the variance matrix was neither asymmetric nor singular. In addition, for smoking and regular exercise, the GBTM has a limitation in that it cannot accurately distinguish between subjects who answered “Yes” at least once during the observation period and those who consistently answered “no” for all 10 health checkups. Therefore, we stratified the subjects based on their responses to smoking and regular exercise during the initial health checkup. For missing values of the lifestyle behavior variables in the 10 health checkup datasets, trajectory patterns were estimated under the assumption that the data were missing at random ^(18)^.

Next, we conducted a multitrajectory model incorporating all three lifestyle behaviors, categorizing the respondents into groups with similar trajectories ^(19)^. We defined a healthy lifestyle (Group 0) as consisting of 449 subjects who were nonsmokers, non-daily drinkers, and regular exercisers throughout the observation period. However, the multitrajectory model may not accurately distinguish Group 0 from the final cohort in the analysis. Hence, we applied the multitrajectory model to subjects other than those in Group 0. In addition, we calculated descriptive statistics using demographic variables such as sex, age, lifestyle, intention to improve lifestyle, and medication history at the initial health checkup for each multitrajectory pattern.

In the third phase, we determined whether multitrajectory patterns contributed to longitudinal changes in health conditions (BMI, sBP, LDL-cholesterol, and HbA1c) over 10 health checkups using a linear mixed-effects model with restricted maximum likelihood estimation. The fixed effects included the trajectory group; number of health checkups; interaction terms of the trajectory group and number of health checkups; age at the initial health checkup; sex; total number of health checkups; being well-rested through sleep (yes/no); use of medications for hypertension, dyslipidemia, and diabetes (yes/no); and medical history of cerebrovascular diseases, cardiovascular diseases, and renal failure or dialysis (yes/no). The number of health checkups incorporates a time element where “3” corresponds to the third health checkup. The interaction term between the trajectory group and the number of checkups was used to determine whether the change in the objective variable over time differed by trajectory group. The total number of health checkups ranged from 10 to 14 owing to the 14-year observation period. Three medication history items were introduced as adjustment variables as they can affect sBP, HbA1c, and LDL-cholesterol among the objective variables. Information on these three medications and medical history was obtained from a self-administered questionnaire completed during the health checkups. The random effect of the intercept and residual for a variable that nested the trajectory group and subject ID (e.g., “trajectory groups - subject ID”) was included.

For all analyses, we set the significance level at 0.05 and conducted two-tailed tests. The Stata 16.0 (Stata Corp LP, TX, USA) software was used for the analysis.

## Results

**Figure 2.**
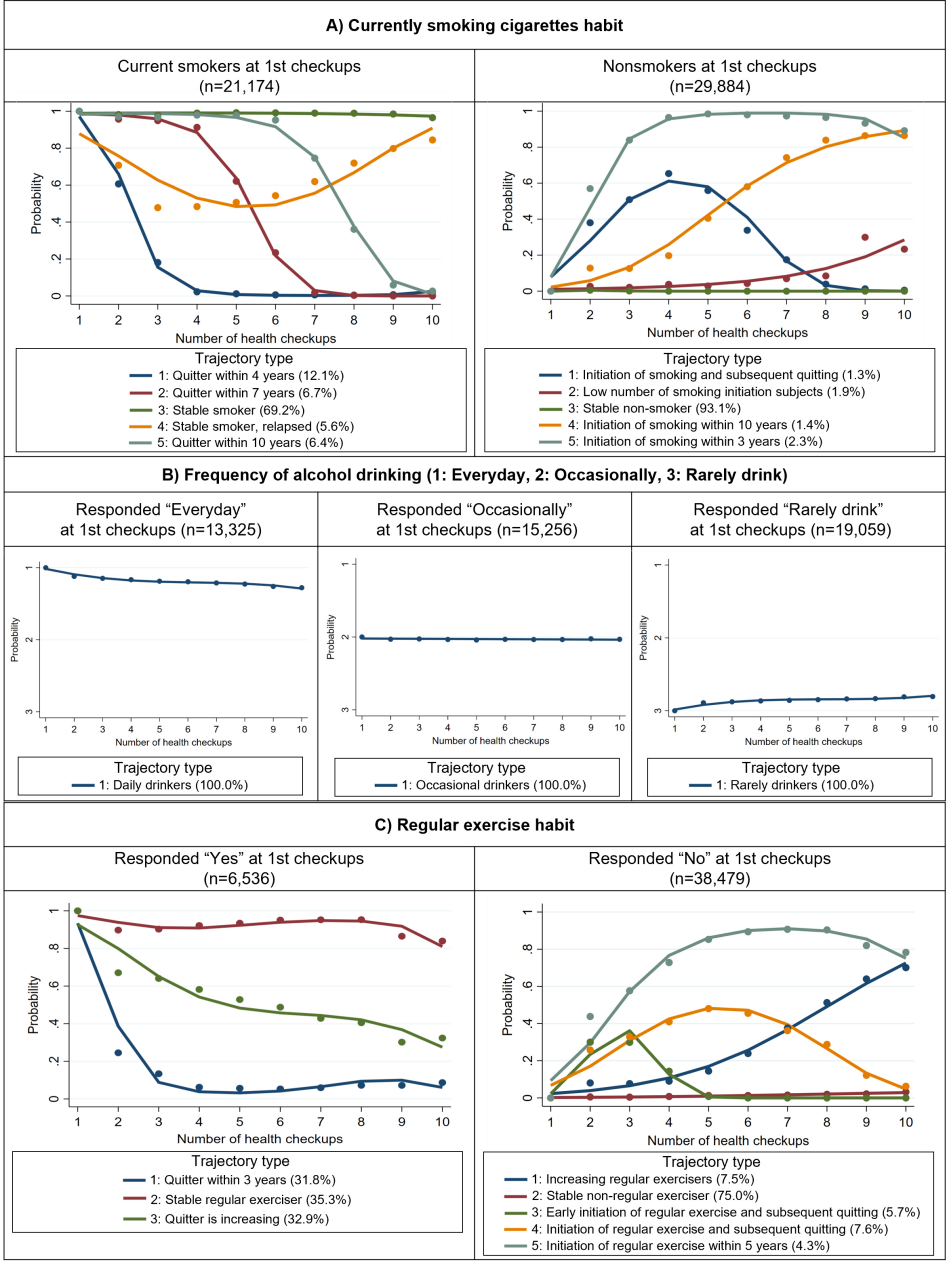
Single trajectories for each lifestyle behavior. A) shows the current smoking habits, B) indicates the frequency of alcohol drinking, and C) indicates regular exercise habits. We used a logit model because A) and C) were binary variables. The closer it is to 1, the greater the probability of the behavior, and vice versa for 0. B) used a Poisson regression model as it is a categorical variable. The y-axis represents the probability of being in each category. The “%” shown in the plot legend indicates the percentage of subjects belonging to each trajectory.

Regarding the trajectory pattern of smoking habits, it was found that 74.8% of current smokers (Trajectory types 3 and 4 on the left side of Figure 2 [A]) and 93.1% of nonsmokers (Trajectory type 3 on the right side of Figure 2 [A]) at the initial health checkup maintained their respective smoking status through the 10th health checkup. In terms of frequency of alcohol consumption, no changes in the status were observed during the observation period (Figure 2 [B]). As for regular exercise habits, only 35.3% of the subjects who initially responded “Yes” continued their exercise habits until the 10th health checkup (Trajectory type 2 on the left side of Figure 2 [C]). Conversely, among those who answered “No” at the initial health checkup, only 11.8% (Trajectory types 1 and 5 on the right side of Figure 2 [C]) adopted a regular exercise regimen by their 10th health checkup.

**Figure 3.**
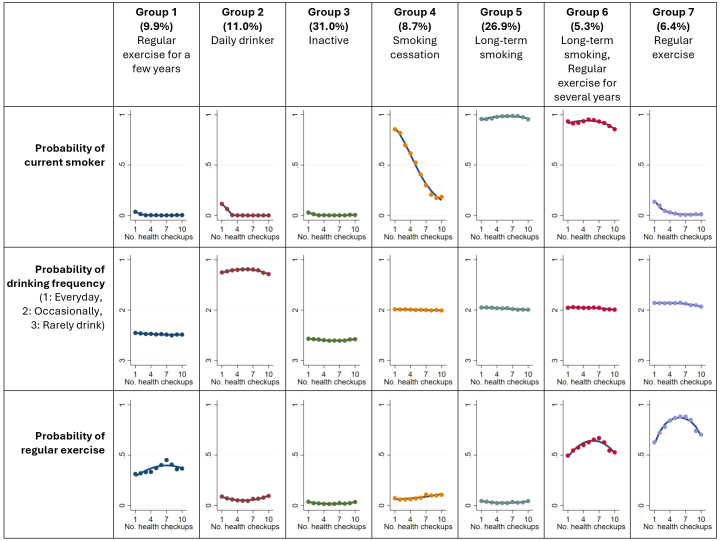
Multitrajectories of current smoking, frequency of alcohol consumption, and regular exercise. We applied the multitrajectory model to subjects other than those in Group 0 (nonsmokers, non-daily drinkers, and regular exercisers from the 1st to the 10th checkup). As a result, 2,423 subjects had no trajectory data in one or more models. This figure arranges the graph by health behavior in the rows and by the trajectory pattern group identified in this analysis in the columns. The y-axis of each plot represents the probability of a health behavior. We used a logit model as current smoking and regular exercise are binary variables. The closer to 1, the greater the probability of the behavior, and vice versa for 0. A Poisson regression model was used for drinking as it is a categorical variable. The y-axis denotes the probability of being in each category. As an example of interpretation, Group 1, which represents 9.9% of the analyzed population, tended to be almost all nonsmokers throughout the observation period. Approximately 40% of the subjects had regular exercise habits at the 4^th^ checkup.

In the multitrajectory model analysis, except for Group 0, Healthy lifestyle (0.9%) subjects were clustered into the following groups: Group 1 - Regular exercise for a few years (9.9%); Group 2 - Daily drinker (11.0%); Group 3 - Inactive (31.0%); Group 4 - Smoking cessation (8.7%); Group 5 - Long-term smoking (26.9%); Group 6 - Long-term smoking, regular exercise for several years (5.3%); and Group 7 - Regular exercise (6.4%) (Figure 3 and Supplementary Table 1 for the description of each trajectory group). Group 0 had a higher proportion of women than the other groups (Table 1). The 40-49 age group consistently represented the largest segment across all trajectory groups. Group 2 had 78.3% of subjects reporting daily drinking habits, whereas Groups 4, 5, 6, and 7 showed distributions of approximately 30% each, spanning daily, occasional, none, or infrequent habits. Regarding regular exercise habits, even in Group 7, which had the highest proportion of subjects answering “Yes” among the groups other than Group 0, the rate was still approximately half. Additional characteristics are listed in Supplementary Table 2. Group 0 had a higher proportion of women than the other groups (Table 1). The 40-49 age group consistently represented the largest segment across all trajectory groups. Group 2 had 78.3% of subjects reporting daily drinking habits, whereas Groups 4, 5, 6, and 7 exhibited distributions of approximately 30% each, spanning daily, occasional, none, or infrequent habits. Regarding regular exercise habits, even in Group 7, which had the highest proportion of subjects answering “Yes” among the groups other than Group 0, the rate was still approximately half. Additional characteristics are presented in Supplementary Table 2.

**Table 1.** Characteristics of the First Health Checkup Grouped by Multitrajectory Modeling.

Variables, %	All (n = 51,064)	Trajectory group							
		Healthy lifestyle (Group 0, n = 449)	Regular exercise for a few years (Group 1, n = 5,062)	Daily drinker (Group 2, n = 5,607)	Inactive (Group 3, n = 15,803)	Smoking cessation (Group 4, n = 4,418)	Long-term smoking (Group 5, n = 13,738)	Long-term smoking, regular exercise for several years (Group 6, n = 2,725)	Regular exercise (Group 7, n = 3,262)
Sex, n (%)							
Male	84.1	55.0	73.1	93.3	70.0	93.3	96.2	95.8	85.0
Female	15.9	45.0	26.9	6.7	30.0	6.7	3.8	4.2	15.0
Age (years)							
20-29	17.1	8.9	18.7	7.3	21.2	19.8	16.8	17.3	10.6
30-39	28.8	16.0	27.6	24.7	32.3	28.5	30.2	24.6	21.1
40-49	41.7	57.2	42.2	50.3	37.7	38.5	41.5	43.8	47.7
50-59	12.3	17.8	11.4	17.6	8.8	13.2	11.6	14.3	20.5
Frequency of drinking							
Daily	26.1	0.0	6.8	78.3	3.0	30.1	34.7	31.7	35.2
Occasional	29.9	38.3	31.1	18.0	34.3	33.0	27.8	27.8	32.1
None or rarely	37.3	61.7	47.8	1.0	57.9	30.0	31.5	29.0	21.5
Missing	6.7	0.0	14.3	2.7	4.7	6.9	5.9	11.4	11.2
Current smoker							
No	58.5	100.0	96.5	88.2	97.2	15.0	4.2	6.7	86.3
Yes	41.5	0.0	3.5	11.8	2.8	85.0	95.8	93.3	13.7
Missing	0.0	0.0	0.0	0.1	0.0	0.0	0.0	0.0	0.0
Exercise at least 2 days a week for at least 30 min							
No	75.4	0.0	57.7	84.9	84.1	80.2	85.5	43.0	31.9
Yes	12.8	100.0	27.3	8.3	2.8	6.5	3.9	42.6	55.6
Missing	11.8	0.0	15.0	6.9	13.0	13.3	10.6	14.5	12.5
Improvement of lifestyle							
I do not intend to improve	13.7	29.0	12.5	10.1	12.6	11.7	15.9	18.2	14.2
I intend to improve (generally within 6 months)	29.9	14.0	25.5	31.6	33.3	30.3	31.6	22.3	18.6
I intend to improve in the near future (generally within a month) and have begun to do so gradually	6.8	9.1	9.5	4.8	7.5	5.9	5.8	7.8	6.2
Already working on improvement (less than 6 months)	3.8	4.7	6.4	2.5	3.7	3.3	2.7	5.8	5.7
Already working on improvement (more than 6 months)	4.3	20.0	6.6	4.2	3.1	3.3	1.9	7.7	13.2
Missing	41.6	23.2	39.5	46.9	39.8	45.5	42.1	38.1	42.1

Data are shown as % in column. The calendar year of the first checkup differed for each subject. The number of subjects in each calendar year was 157 in 2007, 3669 in 2008, 45494 in 2009, 1733 in 2010, and 11 in 2011.

Regarding health condition variables, all four variables (BMI, sBP, LDL-cholesterol, and HbA1c) exhibited a significant upward trend over the observation period (Table 2 and Supplementary Table 3). In Groups 3 and 5, which represent the largest and second-largest trajectory groups, elevated levels of sBP (Group 3: β = 1.18, standard error [SE] = 0.60, p = 0.05; Group 5: β = 1.33, SE = 0.61, p < 0.05) and LDL-cholesterol (Group 3: β = 3.80, SE = 1.36, p < 0.05; Group 5: β = 3.04, SE = 1.37, p < 0.05) were observed compared with those in Group 0. However, there were no clearly increased regression coefficients for the interaction terms between the number of health checkups (1-10) and trajectory group (0-7). Group 2 exhibited elevated sBP estimates (β = 2.43, SE = 0.62, p < 0.001) and an interaction effect (β = 0.15, SE = 0.05, p < 0.05). Moreover, each group showed significant differences in regression coefficients for at least one of the four health condition variables.

**Table 2.** Liner Mixed-Effect Model Estimates for Anthropometric and Laboratory over the Course of 10 Health Checkups.

Parameters	BMI	sBP	LDL-cholesterol	HbA1c
Fixed effect				
Intercept	21.47 (0.24) *	106.25 (0.87) *	81.28 (2.02) *	4.80 (0.04) *
Trajectory group				
Group 0: Healthy lifestyle	Reference	Reference	Reference	Reference
Group 1: Regular exercise for a few years	0.67 (0.17) *	1.37 (0.63) *	4.39 (1.40) *	−0.01 (0.03)
Group 2: Daily drinker	0.02 (0.16)	2.43 (0.62) *	0.29 (1.40)	−0.13 (0.03) *
Group 3: Inactive	0.17 (0.16)	1.18 (0.60)	3.80 (1.36) *	−0.03 (0.03)
Group 4: Smoking cessation	0.07 (0.17)	0.79 (0.63)	3.07 (1.42) *	−0.04 (0.03)
Group 5: Long-term smoking	0.04 (0.16)	1.33 (0.61) *	3.04 (1.37) *	−0.03 (0.03)
Group 6: Long-term smoking, regular exercise for several years	0.57 (0.17) *	1.19 (0.65)	2.85 (1.47)	−0.02 (0.03)
Group 7: Regular exercise	0.30 (0.17)	1.41 (0.64) *	−0.58 (1.44)	−0.09 (0.03) *
No. of health checkups ^a^	0.05 (0.00) *	0.39 (0.05) *	1.35 (0.08) *	0.00 (0.00) *
Interaction of No. of health checkups × Trajectory group				
Group 0: Healthy lifestyle	Reference	Reference	Reference	Reference
Group 1: Regular exercise for a few years	0.03 (0.01) *	−0.06 (0.05)	−0.33 (0.09) *	0.01 (0.00) *
Group 2: Daily drinker	0.03 (0.00) *	0.15 (0.05) *	−0.72 (0.09) *	0.01 (0.00) *
Group 3: Inactive	0.06 (0.00) *	0.01 (0.05)	−0.26 (0.08) *	0.01 (0.00) *
Group 4: Smoking cessation	0.11 (0.01) *	0.20 (0.05) *	−0.40 (0.09) *	0.01 (0.00) *
Group 5: Long-term smoking	0.05 (0.00) *	0.06 (0.05)	−0.74 (0.08) *	0.01 (0.00) *
Group 6: Long-term smoking, regular exercise for several years	0.02 (0.01) *	0.07 (0.05)	−0.84 (0.09) *	0.01 (0.00) *
Group 7: Regular exercise	0.00 (0.01)	0.06 (0.05)	−0.51 (0.09) *	0.00 (0.00)
Age at the first health checkup	0.03 (0.00) *	0.31 (0.01) *	0.98 (0.01) *	0.02 (0.00) *
Women sex	−1.86 (0.04) *	−9.09 (0.15) *	−7.34 (0.35) *	−0.10 (0.01) *
Total No. of health checkups during the observation period ^b^	0.00 (0.02)	0.25 (0.06) *	−0.44 (0.13) *	0.01 (0.00) *
Being well-rested through sleep	0.03 (0.00) *	0.25 (0.04) *	0.42 (0.07) *	−0.01 (0.00) *
Use of antihypertensive medication	0.18 (0.01) *	−4.68 (0.10) *	−5.41 (0.18) *	0.06 (0.00) *
Use of insulin injections or glucose-lowering medication	−0.45 (0.02) *	0.02 (0.18)	−9.33 (0.30) *	0.24 (0.01) *
Use of cholesterol-lowering medication	0.17 (0.01) *	−0.37 (0.11) *	−26.76 (0.18) *	0.06 (0.00) *
Medical history of cerebrovascular disease	−0.20 (0.04) *	−1.49 (0.45) *	−5.56 (0.75) *	−0.04 (0.02) *
Medical history of cardiovascular disease	−0.11 (0.03) *	−1.70 (0.31) *	−12.52 (0.53) *	0.00 (0.01)
Medical history of renal failure or dialysis	−0.06 (0.07)	−1.23 (0.70)	2.02 (1.19)	−0.07 (0.03) *
Random effect
Trajectory group × Subject ID ^c^
Standard deviation of intercept	3.27 (0.01)	10.74 (0.04)	26.00 (0.09)	0.45 (0.00)
Standard deviation of residual	0.89 (0.00)	9.19 (0.01)	15.36 (0.02)	0.31 (0.00)

Data are expressed as coefficients (standard error). Adjusted for age at the first health checkup, sex, total number of health checkups during the observation period, being well-rested through sleep, use of antihypertensive medication, insulin injections or glucose-lowering medication, cholesterol-lowering medication, and medical history of cerebrovascular disease, cardiovascular disease, renal failure, or dialysis. Abbreviations: BMI, body mass index; sBP, systolic blood pressure; HbA1c, hemoglobin A1c * p-value < 0.05. ^a^ Number of health checkups incorporates a time element, where “3” corresponds to the third health checkup. ^b^ Total number of health checkups ranged from 10 to 14 owing to the 14-year observation period. ^c^ We created a variable by nesting the “trajectory group” and “Subject ID” (e.g., “trajectory groups − subject ID”) and used it as a random effect.

## Discussion

In this study, we examined the trajectories of lifestyle behaviors in Japanese adults using health checkup data and analyzed subsequent changes in health conditions. We identified seven trajectory groups in addition to Group 0, which represented a healthy lifestyle. Notably, higher percentages of the population analyzed represented Group 3: Inactive (31.0%), Group 5: Long-term smoking (26.9%), and Group 2: Daily drinker (11.0%). All trajectory groups showed significantly higher values of at least one health condition, such as BMI, sBP, LDL-cholesterol, and HbA1c, than Group 0 during the observation period. Specifically, Group 5 had significantly higher sBP and LDL-cholesterol levels than Group 0 throughout the observation period. Furthermore, Group 2 had significantly higher sBP. This study is the first to investigate the trajectories of three combined health behaviors in Japanese adults, providing insights into the benefits of long-term healthy lifestyles, including regular exercise as well as abstinence from smoking and drinking. In particular, the results indicated that promoting regular physical activity among the “inactive” population, which represents 31.0% of the analyzed population, has substantial public health implications in terms of disease prevention.

In the long-term smoking group (Group 5), although sBP and LDL-cholesterol levels did not exhibit an increase in the interaction effect over time, they were still higher than those in Group 0 during the observation period. The potential mechanism for increased sBP among smokers is believed to be stimulation of the sympathetic nervous system and increased blood pressure because of nicotine intake from tobacco ^(20)^. Contrarily, increased lipid levels in smokers may be due to tissue lipolysis induced by the catecholamine and adenyl cyclase axes ^(21)^. However, it is noteworthy that a cross-sectional study conducted in the USA reported no significant differences in LDL-cholesterol levels between smokers and nonsmokers ^(22)^, which contradicts the findings of this study. Nonsmoking groups, such as Group 1, also had significantly higher LDL-cholesterol levels than Group 0. The significant increase observed in this study is likely due to differences in physical activity and unmeasured confounders, such as fat or dietary fiber intake, in addition to the effects of tobacco smoking ^(23), (24), (25), (26), (27)^.

The highest percentage of the inactive group (Group 3) is consistent with the findings of two previous studies―a cohort of Taiwanese adults aged 65 years and above and women in a cross-sectional study of Japanese adults ^(10), (28)^. Throughout the observation period, Group 3 consistently exhibited higher LDL-cholesterol levels than Group 0. In the interaction effect over time, compared with Group 0, Group 3 showed increasing trends in BMI and HbA1c while experiencing a declining trend in LDL-cholesterol. Group 3 comprised individuals without smoking or drinking habits, suggesting that the only lifestyle factor that differed from Group 0 was physical inactivity. Therefore, the effects of insufficient physical activity on health conditions were clearly observed in the results. Physical activity was found to be preventive for all four outcomes ^(23), (24), (25), (29), (30), (31)^. Physical activity may lower blood pressure through mechanisms, such as reduced age-related endothelial dysfunction and increased insulin sensitivity ^(32)^. For LDL-cholesterol, aerobic exercise has been suggested to also affect the levels of adiponectin, which is one of the adipocytokines involved in dyslipidemia ^(33)^. In addition, exercise increases glucose uptake in the skeletal muscles and lowers blood glucose levels ^(34)^, contributing to improved HbA1c levels. One possible reason for the increase in BMI observed in this study is the imbalance between dietary energy intake and expenditure resulting from physical inactivity ^(35)^.

Group 2, the daily drinking group, had higher sBP and lower HbA1c levels than Group 0. Group 2 exhibited increasing trends in BMI, sBP, and HbA1c and a decreasing trend in LDL-cholesterol compared with Group 0. Previous meta-analyses and Japanese cohort studies have reported that drinkers have a higher risk of hypertension than nondrinkers ^(36), (37)^. The mechanisms by which alcohol induces hypertension are diverse, but one contributing factor is endothelial dysfunction associated with alcohol consumption ^(38)^. The association between alcohol consumption and HbA1c level is complex, with some studies suggesting a U-shaped association between alcohol consumption (g/day) and the risk of incident type 2 diabetes ^(39)^. However, a Japanese systematic review including seven cohort studies demonstrated that moderate drinking increased the risk of type 2 diabetes in men with BMI ≤ 22 ^(40)^. In our study, due to a high proportion of missing values for the amount of alcohol consumed during the initial health checkup (Supplementary Table 2), only the frequency of alcohol consumption could be utilized in the analysis. Further research is warranted to examine the association between the trajectory patterns of alcohol consumption and HbA1c levels.

Regarding exercise, the trajectory groups, shown in Figure 3, were characterized by regular exercise (Group 0, 0.9%; Group 7, 6.4%) and represented a small percentage of the analyzed population. In Japan, the lack of discretionary time for regular exercise due to work commitments is a barrier to ^(41), (42)^ physical activity. In terms of drinking frequency, no trajectory groups that showed changes in drinking habits were identified. Genetic factors may play a role in the drinking habits of the Japanese population as they have a higher percentage of ALDH2*2 allele carriers, which can cause facial flushing, increased heart rate, and nausea when drinking alcohol compared with Caucasians ^(43)^. This genetic background may make Japanese individuals less likely to change their drinking habits ^(44)^. We also observed a decreasing trend in LDL-cholesterol in all groups compared with Group 0. Sex differences in age-related changes in LDL-cholesterol ^(45)^ may have influenced this association as LDL-cholesterol tends to increase after menopause. The higher proportion of women in Group 0 than in the other groups may have contributed to this finding. However, negative regression coefficients persisted for Groups 2, 5, and 6 when stratified by sex (Supplementary Table 4). The factors that influence LDL-cholesterol changes include dietary fiber intake ^(46)^. Therefore, the results may be due to several unmeasured factors, including dietary intake.

For the other trajectory groups, failure to practice any of the health behaviors led to worse health conditions compared with Group 0. Groups 1 (regular exercise for few years) and 7 (regular exercise) included individuals who were slightly smokers and habitual drinkers, respectively. Group 1 exhibited significantly higher LDL-cholesterol levels, sBP, and BMI, whereas Group 7 showed significantly higher sBP. Group 4 (smoking cessation) was the only group in the multitrajectory model that showed a change in health behaviors. After smoking cessation, Group 4 exhibited similar health behavior trends to Group 3 (inactive), and both groups had significantly higher LDL-cholesterol levels than Group 0. The differences in health conditions compared with Group 0 could be attributed to the effects of physical activity. In addition, physical activity was positively correlated with health literacy ^(47)^. A Japanese cross-sectional study found that the number of health behaviors practiced was significantly higher in the group with higher health literacy than in the group with low health literacy ^(48)^. Further research is warranted to determine the effects of lifestyle behavioral patterns other than smoking, frequency of alcohol consumption, and regular exercise on health conditions.

The strengths of this study are its large sample size and long follow-up period, with data from 10 or more health checkups. However, this study has several limitations that need to be acknowledged. First, there was a significant gender imbalance in the study population, with 84% of the subjects being men. This may underestimate the effects of smoking on health conditions in women as the smoking rates in women are lower than those in men in Japan. Second, the study population mainly consisted of individuals from large-scale enterprises, which may have introduced selection bias and limited the generalizability of the results to a broader population. Moreover, the lack of information on socioeconomic status in the databases used for the study prevented the control of its potential confounding effect. Third, the health checkup questionnaire did not include information on diet, nutritional intake, as well as intensity and type of physical activity, which could be a potential confounding factor. The absence of such data may have resulted in some unmeasured confounding factors in the analysis of lifestyle behaviors and health conditions.

In conclusion, we identified seven trajectory patterns of lifestyle behaviors in addition to the healthy lifestyle group (Group 0) using smoking, drinking frequency, and regular exercise habits as factors. However, it should be noted that the proportion of individuals who quit smoking, changed their drinking habits, or exercised regularly was relatively low. Failure to adopt these healthy behaviors was associated with inferior health conditions compared with those in Group 0. Regular exercise and abstinence from smoking and drinking may be essential to avoid deterioration of health conditions.

## Article Information

### Conflicts of Interest

None

### Sources of Funding

This study was supported by the National Center Cohort Collaborative for Advancing Population Health (Project Number 2019-(1)-1) and Research for Predictive Preemptive Medicine Using Pre-medical Lifelog Data and Health Checkup (Project Number 2020-B-08) funded by the Japan Health Research Promotion Bureau (JH) Research Fund.

### Acknowledgement

We would like to thank Editage (www.editage.jp) for English language editing.

### Author Contributions

TT, RK, JU, and MI conceived and designed the study. TT, RK, MF, ST, and YO performed the data analysis and interpretation. TT analyzed the data and wrote the first draft of the manuscript. All authors reviewed and interpreted the results and approved the final version of the manuscript.

### Approval by Institutional Review Board (IRB)

The Japanese ethical guidelines “Ethical Guidelines for Medical and Health Sciences Research Involving Human Subjects” (Chapter 1: General Provisions, Section 3: Scope) do not apply to this study as it uses anonymized processed information created by JMDC, Inc.

## Supplement

Supplementary Table 1

Supplementary Table 2

Supplementary Table 3

Supplementary Table 4

Supplementary Figure 5.1, Supplement Figure 5.2, Supplement Table 5.1, Supplement Table 5.2
