# Supplementary material for: Trajectory Patterns of Three Lifestyle Behaviors and Subsequent Health Conditions in Japanese Adults: A Retrospective Longitudinal Study Using a Health Checkup Database
Source: JMA J. 2024 Oct 3;7(4):506–17. doi: 10.31662/jmaj.2024-0076 (PMC11543322; doi:10.31662/jmaj.2024-0076)
Supplement: Supplementary Table 1 [file 2433-3298-7-4-0506-s001.pdf]

Supplementary Table 1. Description of the health lifestyle and seven trajectory groups clustered by multitrajectory modeling.

Trajectory group	Description
Group 0: Healthy lifestyle	Non-smokers, non-daily drinkers, and regular exercisers during the observation period.
Group 1: Regular exercise for a few years	Long-term non-smokers and regular exercised for few years during the observation period. This group included about equal proportions of occasional drinkers and rarely drinkers.
Group 2: Daily drinker	Long-term non-smokers, daily drinkers, and non-exercisers.
Group 3: Inactive	Long-term non-smokers and non-exercisers. This group included about equal proportions of occasional drinkers and rarely drinkers.
Group 4: Smoking cessation	Subjects who quit smoking during the observation period and long-term non-exercisers. This group included about equal proportions of daily, occasional, and rarely drinkers.
Group 5: Long-term smoking	Long-term smokers and non-exercisers. This group included about equal proportions of daily, occasional, and rarely drinkers.
Group 6: Long-term smoking, regular exercise for several years	Long-term smokers and regular exercise for several years during the observation period. This group included about equal proportions of daily, occasional, and rarely drinkers.
Group 7: Regular exercise	Long-term non-smokers. This group included about equal proportions of daily, occasional, and rarely drinkers. More subjects in this group continued to exercise during the observation period.
