# Supplementary material for: Trajectory Patterns of Three Lifestyle Behaviors and Subsequent Health Conditions in Japanese Adults: A Retrospective Longitudinal Study Using a Health Checkup Database
Source: JMA J. 2024 Oct 3;7(4):506–17. doi: 10.31662/jmaj.2024-0076 (PMC11543322; doi:10.31662/jmaj.2024-0076)
Supplement: Supplementary Table 2 [file 2433-3298-7-4-0506-s002.pdf]

Supplementary Table 2. Characteristics of the first health checkup (items not included in Table 1) grouped by multitrajectory modeling.

Variables, %	All (n=51,064)	Trajectory group							
		Healthy lifestyle (Group 0, n=449)	Regular exercise for a few years (Group 1, n=5,062)	Daily drinker (Group 2, n=5,607)	Inactive (Group 3, n=15,803)	Smoking cessation (Group 4, n=4,418)	Long-term smoking (Group 5, n=13,738)	Long-term smoking, regular exercise for several years (Group 6, n=2,725)	Regular exercise (Group 7, n=3,262)
Eating speed									
Fast	13.8	20.0	15.2	10.4	12.7	14.0	14.4	18.1	15.4
Normal	31.9	46.8	33.6	28.7	33.0	28.3	31.5	34.6	31.6
Slow	13.6	10.9	12.6	14.9	15.3	12.9	12.9	10.0	11.9
Missing	40.8	22.3	38.6	46.0	39.1	44.8	41.3	37.2	41.1
Late dinner									
No	40.1	63.7	46.9	32.2	45.8	35.5	35.5	38.3	39.4
Yes	19.2	14.3	14.5	21.8	15.1	19.7	23.2	24.5	19.7
Missing	40.7	22.0	38.5	46.0	39.1	44.8	41.3	37.2	41.0
Late snacking									
No	68.5	77.5	65.8	74.5	64.3	68.2	70.7	69.6	71.1
Yes	19.5	22.5	19.3	18.6	22.5	18.3	18.3	15.5	16.3
Missing	12.0	0.0	14.9	6.9	13.2	13.5	11.0	14.9	12.6
Skipping breakfast									
No	71.2	95.3	76.5	79.3	75.1	65.8	61.9	63.0	79.5
Yes	16.7	4.7	8.5	13.7	11.7	20.6	27.0	22.0	7.8
Missing	12.1	0.0	15.0	7.0	13.2	13.5	11.0	15.0	12.7
Amount of alcohol									

<20 g	20.8	27.8	20.0	14.4	23.9	20.8	19.5	23.3	20.1
20-40 g	9.9	5.6	6.1	15.3	5.6	10.5	12.2	16.4	11.9
40-60 g	3.7	1.3	1.9	5.8	1.7	4.0	4.9	5.9	4.7
>60 g	1.3	0.9	0.6	1.2	0.7	1.4	1.8	2.3	1.6
Missing	64.4	64.4	71.4	63.3	68.1	63.3	61.5	52.1	61.7
At least 1 hour per day of walking or equivalent physical activity									
No	70.1	46.1	60.5	81.2	71.5	72.0	74.3	53.0	56.4
Yes	17.8	53.9	24.5	11.8	15.3	14.4	14.6	32.0	30.8
Missing	12.1	0.0	15.0	7.0	13.2	13.5	11.1	15.0	12.8
Walking fast									
No	29.9	26.1	27.5	25.4	33.6	28.1	32.4	25.9	19.5
Yes	23.7	50.1	29.5	21.8	21.2	21.4	20.5	33.4	35.3
Missing	46.3	23.8	43.0	52.8	45.1	50.4	47.1	40.7	45.2
Being well-rested through sleep									
No	24.7	19.6	21.9	23.3	26.0	24.1	26.8	23.2	18.7
Yes	34.2	57.5	38.9	30.6	34.5	30.9	31.8	39.2	39.6
Missing	41.1	22.9	39.2	46.1	39.6	45.0	41.3	37.6	41.7
Increased more than 10 kg from the weight at age 20									
No	43.3	66.1	45.6	36.8	46.6	38.5	41.3	44.4	45.0
Yes	16.0	11.8	15.9	17.2	14.2	16.7	17.4	18.3	14.0
Missing	40.8	22.0	38.6	46.0	39.2	44.8	41.3	37.3	41.0
Weight gain or loss of more than ± 3 kg in the past year									
No	73.2	87.5	69.7	80.6	72.9	70.9	73.7	66.3	72.6
Yes	14.2	11.4	14.6	12.2	13.1	14.9	15.1	18.4	14.4

Missing	12.6	1.1	15.7	7.2	14.0	14.2	11.2	15.3	13.0
Hypertension drug use									
No	97.5	98.0	97.2	96.1	98.2	97.3	97.8	97.3	96.3
Yes	2.5	2.0	2.8	3.9	1.8	2.7	2.2	2.7	3.7
Missing	0.0	0.0	0.0	0.0	0.0	0.0	0.0	0.0	0.0
Diabetes drug use									
No	99.4	99.1	99.3	99.7	99.5	99.5	99.2	99.0	99.3
Yes	0.6	0.9	0.7	0.3	0.5	0.5	0.8	1.0	0.7
Missing	0.0	0.0	0.0	0.0	0.0	0.0	0.0	0.0	0.0
Dyslipidemia drug use									
No	97.1	97.3	97.5	96.8	97.3	96.9	96.7	97.1	97.2
Yes	2.9	2.7	2.5	3.2	2.7	3.1	3.3	2.9	2.8
Missing	0.0	0.0	0.0	0.0	0.0	0.0	0.0	0.0	0.0
Eligibility information of health insurance									
Himself/herself	92.8	61.2	83.1	97.8	87.9	97.5	99.2	98.3	90.0
Family member	7.2	38.8	16.9	2.2	12.1	2.5	0.8	1.7	10.0

Data are shown as % in column.

The calendar year of the first checkup differed for each subject. The number of subjects in each calendar year was 157 in 2007, 3669 in 2008, 45494 in 2009, 1733 in 2010, and 11 in 2011.
