# Supplementary material for: Trajectory Patterns of Three Lifestyle Behaviors and Subsequent Health Conditions in Japanese Adults: A Retrospective Longitudinal Study Using a Health Checkup Database
Source: JMA J. 2024 Oct 3;7(4):506–17. doi: 10.31662/jmaj.2024-0076 (PMC11543322; doi:10.31662/jmaj.2024-0076)
Supplement: Supplementary Table 3 [file 2433-3298-7-4-0506-s003.pdf]

Supplementary Table 3. Mean and standard deviation of health condition variables by trajectory group over the course of 10 health checkups.

Trajectory group	No. of health checkups									
	1	2	3	4	5	6	7	8	9	10
BMI										
Group 0: Healthy lifestyle	22.09 (3.18)	22.13 (3.19)	22.21 (3.17)	22.2 (3.2)	22.25 (3.2)	22.29 (3.24)	22.32 (3.19)	22.47 (3.27)	22.53 (3.31)	22.54 (3.26)
Group 1: Regular exercise for a few years	22.9 (3.41)	23 (3.45)	23.14 (3.51)	23.17 (3.52)	23.25 (3.56)	23.29 (3.61)	23.38 (3.63)	23.5 (3.64)	23.62 (3.66)	23.67 (3.68)
Group 2: Daily drinker	22.81 (2.87)	22.91 (2.9)	23.07 (2.91)	23.07 (2.92)	23.15 (2.93)	23.21 (2.95)	23.29 (2.98)	23.4 (3.02)	23.57 (3.05)	23.56 (3.08)
Group 3: Inactive	22.45 (3.49)	22.53 (3.52)	22.69 (3.56)	22.75 (3.6)	22.84 (3.63)	22.92 (3.66)	23.03 (3.7)	23.17 (3.76)	23.34 (3.79)	23.37 (3.81)
Group 4: Smoking cessation	22.93 (3.3)	23.01 (3.34)	23.24 (3.38)	23.34 (3.39)	23.5 (3.42)	23.62 (3.45)	23.8 (3.46)	24.01 (3.49)	24.19 (3.51)	24.23 (3.54)
Group 5: Long-term smoking	22.85 (3.39)	22.89 (3.41)	23.07 (3.44)	23.11 (3.46)	23.18 (3.48)	23.25 (3.51)	23.35 (3.53)	23.49 (3.56)	23.65 (3.59)	23.71 (3.61)
Group 6: Long-term smoking, regular exercise for several years	23.37 (3.3)	23.39 (3.29)	23.55 (3.35)	23.53 (3.36)	23.57 (3.35)	23.6 (3.35)	23.66 (3.38)	23.81 (3.42)	23.96 (3.47)	24.04 (3.49)
Group 7: Regular exercise	22.95 (3.05)	23 (3.05)	23.09 (3.07)	23.08 (3.06)	23.12 (3.05)	23.14 (3.09)	23.2 (3.12)	23.31 (3.18)	23.44 (3.22)	23.45 (3.25)
sBP										
Group 0: Healthy lifestyle	118.36 (15.36)	119.23 (15.41)	120.2 (16.55)	120.43 (15.71)	120.23 (15.18)	119.96 (15.05)	120.55 (15.97)	120.68 (15.1)	122.22 (16.14)	122.37 (16.26)
Group 1: Regular exercise for a few years	120.17 (14.34)	120.65 (14.76)	121 (14.56)	120.74 (14.48)	120.74 (14.45)	120.89 (14.57)	121.29 (14.82)	121.54 (14.79)	122.98 (15.45)	123.31 (15.74)
Group 2: Daily drinker	124.66 (13.8)	125.49 (14.19)	125.96 (13.73)	125.67 (13.5)	125.66 (13.52)	126.12 (13.54)	126.54 (13.61)	126.78 (13.65)	129.19 (14.66)	129.34 (14.91)
Group 3: Inactive	119.83 (14.27)	120.14 (14.37)	120.86 (14.27)	120.48 (14.33)	120.61 (14.2)	120.82 (14.15)	121.11 (14.31)	121.38 (14.25)	123.1 (15.17)	123.28 (15.6)
Group 4: Smoking cessation	122.03 (13.62)	122.56 (13.62)	123.44 (13.79)	123.42 (14.2)	123.61 (14.1)	123.84 (13.91)	124.39 (14.06)	124.74 (13.69)	126.42 (14.94)	126.63 (15.08)
Group 5: Long-term smoking	122.84 (13.9)	123.25 (13.8)	123.79 (13.7)	123.47 (13.85)	123.51 (13.6)	123.64 (13.82)	124.06 (13.84)	124.61 (14.18)	126.36 (15.05)	126.73 (15.38)
Group 6: Long-term smoking, regular exercise for several years	122.4 (13.64)	123.27 (13.91)	124.09 (13.75)	123.32 (13.63)	123.33 (13.76)	123.95 (13.85)	124.21 (14.17)	124.5 (14.46)	126.23 (15.07)	126.55 (14.9)
Group 7: Regular exercise	122.46 (14.53)	122.9 (14.26)	123.81 (14.31)	123.45 (14.16)	123.73 (14.58)	124.05 (14.6)	124.35 (14.54)	124.3 (15.01)	126.14 (15.33)	126.21 (16.08)
LDL-cholesterol										

Group 0: Healthy lifestyle	117.3 (28.26)	117 (29.16)	119.13 (27.93)	118.86 (27.7)	120.02 (29.79)	122.06 (28.02)	123.45 (29.51)	122.71 (28.37)	124.93 (28.29)	127.07 (29.81)
Group 1: Regular exercise for a few years	118.69 (30.94)	119.11 (30.72)	121.52 (30.44)	120.76 (30.23)	120.31 (30.29)	122.39 (30.53)	122.76 (30.42)	123.57 (29.96)	125.8 (30.29)	125.68 (29.45)
Group 2: Daily drinker	118.22 (30.23)	118.21 (30.88)	120.03 (30.45)	118.78 (29.88)	117.88 (29.68)	119.17 (29.71)	119.73 (29.87)	120.18 (29.69)	121.56 (29.58)	122.1 (29.32)
Group 3: Inactive	117.16 (30.06)	117.12 (30.4)	119.55 (30.26)	118.81 (29.69)	118.68 (29.61)	120.75 (29.8)	121.48 (29.71)	122.26 (29.49)	123.99 (29.37)	124.57 (29.57)
Group 4: Smoking cessation	118.8 (31.76)	118.85 (32.03)	121.57 (32.21)	120.48 (31.23)	120.39 (30.91)	121.85 (31.41)	122.41 (31.22)	122.57 (30.98)	123.08 (30.58)	123.6 (30.59)
Group 5: Long-term smoking	118.66 (31.95)	117.83 (31.87)	120.3 (31.75)	119.45 (31.53)	119.25 (31.02)	120.71 (31.19)	120.73 (31.2)	120.68 (30.8)	121.49 (30.77)	121.66 (30.9)
Group 6: Long-term smoking, regular exercise for several years	119.41 (32.12)	119.37 (31.48)	121.46 (31.55)	120.1 (31.42)	119.22 (30.68)	120.75 (30.83)	120.93 (31.14)	120.49 (30.7)	121.49 (30.23)	121.6 (30.88)
Group 7: Regular exercise	117.84 (30.39)	118.58 (30.36)	119.75 (29.32)	119.65 (29.64)	119.1 (29.84)	120.33 (29.51)	120.45 (29.51)	121.44 (29.55)	122.94 (29.39)	123.22 (28.89)
HbA1c										
Group 0: Healthy lifestyle	5.54 (0.6)	5.59 (0.66)	5.52 (0.64)	5.51 (0.56)	5.54 (0.57)	5.55 (0.71)	5.58 (0.79)	5.55 (0.69)	5.58 (0.65)	5.61 (0.77)
Group 1: Regular exercise for a few years	5.53 (0.65)	5.58 (0.67)	5.55 (0.69)	5.52 (0.66)	5.55 (0.68)	5.57 (0.67)	5.58 (0.69)	5.59 (0.68)	5.61 (0.67)	5.64 (0.65)
Group 2: Daily drinker	5.5 (0.5)	5.54 (0.55)	5.48 (0.57)	5.47 (0.5)	5.48 (0.52)	5.52 (0.51)	5.53 (0.52)	5.56 (0.53)	5.55 (0.49)	5.59 (0.52)
Group 3: Inactive	5.49 (0.57)	5.52 (0.6)	5.47 (0.59)	5.47 (0.58)	5.5 (0.59)	5.52 (0.58)	5.52 (0.59)	5.55 (0.62)	5.57 (0.58)	5.6 (0.59)
Group 4: Smoking cessation	5.55 (0.63)	5.57 (0.61)	5.54 (0.68)	5.54 (0.68)	5.56 (0.66)	5.6 (0.68)	5.61 (0.68)	5.65 (0.7)	5.66 (0.67)	5.69 (0.67)
Group 5: Long-term smoking	5.56 (0.64)	5.58 (0.65)	5.53 (0.67)	5.54 (0.67)	5.56 (0.66)	5.59 (0.66)	5.6 (0.68)	5.63 (0.7)	5.64 (0.64)	5.67 (0.67)
Group 6: Long-term smoking, regular exercise for several years	5.61 (0.68)	5.62 (0.66)	5.58 (0.76)	5.58 (0.69)	5.58 (0.64)	5.62 (0.72)	5.62 (0.69)	5.65 (0.77)	5.68 (0.78)	5.71 (0.75)
Group 7: Regular exercise	5.52 (0.53)	5.55 (0.56)	5.5 (0.57)	5.49 (0.54)	5.49 (0.49)	5.51 (0.5)	5.53 (0.5)	5.55 (0.58)	5.56 (0.51)	5.58 (0.51)

The data represents mean (standard deviation).

Abbreviations: BMI, body mass index; sBP, systolic blood pressure; HbA1c, hemoglobin A1c
