# Supplementary material for: Trajectory Patterns of Three Lifestyle Behaviors and Subsequent Health Conditions in Japanese Adults: A Retrospective Longitudinal Study Using a Health Checkup Database
Source: JMA J. 2024 Oct 3;7(4):506–17. doi: 10.31662/jmaj.2024-0076 (PMC11543322; doi:10.31662/jmaj.2024-0076)
Supplement: Supplementary Table 4 [file 2433-3298-7-4-0506-s004.pdf]

Supplementary Table 4. Liner mixed-effect model estimates for anthropometric and laboratory over the course of 10 health checkups stratified by sex.

Parameters	BMI	sBP	LDL-cholesterol	HbA1c
Sex: men				
Fixed effect				
Intercept	20.99 (0.29) *	105.62 (1.03) *	83.40 (2.47) *	4.71 (0.05) *
Trajectory group				
Group 0: Healthy lifestyle	Reference	Reference	Reference	Reference
Group 1: Regular exercise for a few years	0.81 (0.22) *	2.04 (0.82) *	7.52 (1.90) *	−0.01 (0.04)
Group 2: Daily drinker	0.17 (0.22)	2.66 (0.81) *	3.47 (1.87)	−0.08 (0.04) *
Group 3: Inactive	0.29 (0.21)	1.63 (0.80) *	7.45 (1.85) *	−0.02 (0.04)
Group 4: Smoking cessation	0.18 (0.22)	1.03 (0.81)	5.49 (1.89) *	0.01 (0.04)
Group 5: Long-term smoking	0.14 (0.21)	1.46 (0.80)	5.23 (1.85) *	0.02 (0.04)
Group 6: Long-term smoking, regular exercise for several years	0.71 (0.22) *	1.40 (0.83)	4.89 (1.92) *	0.03 (0.04)
Group 7: Regular exercise	0.43 (0.22)	1.93 (0.83) *	1.99 (1.92)	−0.08 (0.04) *
No. of health checkups [a]	0.05 (0.01) *	0.23 (0.07) *	0.81 (0.12) *	0.01 (0.00) *
Interaction of No. of health checkups × Trajectory group				
Group 0: Healthy lifestyle	Reference	Reference	Reference	Reference
Group 1: Regular exercise for a few years	0.03 (0.01) *	0.02 (0.07)	−0.12 (0.12)	0.00 (0.00)
Group 2: Daily drinker	0.03 (0.01) *	0.28 (0.07) *	−0.27 (0.12) *	0.00 (0.00)
Group 3: Inactive	0.06 (0.01) *	0.08 (0.07)	−0.01 (0.12)	0.01 (0.00)
Group 4: Smoking cessation	0.10 (0.01) *	0.33 (0.07) *	0.04 (0.12)	0.00 (0.00)
Group 5: Long-term smoking	0.05 (0.01) *	0.22 (0.07) *	−0.24 (0.12) *	0.00 (0.00)
Group 6: Long-term smoking, regular exercise for several years	0.02 (0.01) *	0.23 (0.07) *	−0.35 (0.12) *	0.00 (0.00)
Group 7: Regular exercise	0.00 (0.01)	0.19 (0.07) *	−0.16 (0.12)	0.00 (0.00)
Age at 1st health checkup	0.03 (0.00) *	0.28 (0.01) *	0.90 (0.02) *	0.02 (0.00) *
Total No. of health checkups during the observation period [b]	0.04 (0.02)	0.41 (0.06) *	−0.53 (0.15) *	0.01 (0.00) *
Being well-rested through sleep	0.03 (0.00) *	0.28 (0.05) *	0.51 (0.08) *	−0.01 (0.00) *
Use of antihypertensive medication	0.18 (0.01) *	−4.38 (0.11) *	−5.68 (0.19) *	0.07 (0.00) *

Use of insulin injections or glucose-lowering medication	−0.42 (0.02) *	0.26 (0.18)	−9.38 (0.32) *	0.24 (0.01) *
Use of cholesterol-lowering medication	0.19 (0.01) *	−0.36 (0.11) *	−24.24 (0.20) *	0.07 (0.01) *
Medical history of cerebrovascular disease	−0.18 (0.05) *	−1.52 (0.47) *	−5.09 (0.80) *	−0.05 (0.02) *
Medical history of cardiovascular disease	−0.15 (0.03) *	−2.03 (0.33) *	−13.81 (0.56) *	−0.01 (0.01)
Medical history of renal failure or dialysis	−0.02 (0.07)	−1.08 (0.76)	1.42 (1.31)	−0.07 (0.03) *
Random effect				
Trajectory group × Subject ID [c]				
Standard deviation of intercept	3.26 (0.01)	10.35 (0.04)	26.22 (0.10)	0.46 (0.00)
Standard deviation of residual	0.89 (0.00)	9.23 (0.01)	15.60 (0.02)	0.32 (0.00)
Sex: women				
Fixed effect				
Intercept	20.97 (0.51) *	96.23 (1.96) *	50.93 (3.81) *	4.95 (0.06) *
Trajectory group				
Group 0: Healthy lifestyle	Reference	Reference	Reference	Reference
Group 1: Regular exercise for a few years	0.44 (0.26)	0.26 (1.05)	1.34 (1.99)	0.02 (0.03)
Group 2: Daily drinker	−0.50 (0.30)	1.69 (1.22)	−13.62 (2.31) *	−0.19 (0.03) *
Group 3: Inactive	−0.01 (0.25)	0.87 (1.00)	0.62 (1.90)	−0.02 (0.03)
Group 4: Smoking cessation	0.14 (0.32)	−0.52 (1.29)	−1.45 (2.45)	−0.14 (0.04) *
Group 5: Long-term smoking	0.29 (0.29)	0.25 (1.16)	−2.30 (2.21)	−0.16 (0.03) *
Group 6: Long-term smoking, regular exercise for several years	0.09 (0.41)	−1.06 (1.68)	1.38 (3.19)	−0.03 (0.05)
Group 7: Regular exercise	0.11 (0.29)	−0.47 (1.16)	−3.65 (2.21)	−0.03 (0.03)
No. of health checkups [a]	0.05 (0.01) *	0.59 (0.07) *	2.10 (0.11) *	0.00 (0.00)
Interaction of No. of health checkups × Trajectory group				
Group 0: Healthy lifestyle	Reference	Reference	Reference	Reference
Group 1: Regular exercise for a few years	0.02 (0.01) *	−0.05 (0.08)	−0.28 (0.12) *	0.00 (0.00)
Group 2: Daily drinker	0.04 (0.01) *	0.24 (0.09) *	−0.44 (0.14) *	0.01 (0.00) *
Group 3: Inactive	0.06 (0.01) *	0.01 (0.07)	−0.36 (0.11) *	0.00 (0.00) *

Group 4: Smoking cessation	0.12 (0.01) *	0.30 (0.09) *	−0.10 (0.15)	0.01 (0.00) *
Group 5: Long-term smoking	0.04 (0.01) *	−0.02 (0.08)	−0.53 (0.13) *	0.01 (0.00) *
Group 6: Long-term smoking, regular exercise for several years	0.03 (0.01) *	−0.03 (0.12)	−0.47 (0.19) *	0.01 (0.00) *
Group 7: Regular exercise	0.00 (0.01)	0.05 (0.09)	−0.29 (0.13) *	0.00 (0.00)
Age at 1st health checkup	0.04 (0.00) *	0.48 (0.02) *	1.48 (0.04) *	0.01 (0.00) *
Total No. of health checkups during the observation period [b]	−0.13 (0.04) *	−0.30 (0.14) *	−0.15 (0.28)	0.00 (0.00)
Being well-rested through sleep	0.03 (0.01) *	0.20 (0.10) *	0.36 (0.16) *	−0.01 (0.00) *
Use of antihypertensive medication	0.15 (0.03) *	−5.71 (0.31) *	−1.51 (0.49) *	0.03 (0.01) *
Use of insulin injections or glucose-lowering medication	−0.97 (0.07) *	−1.32 (0.72)	−5.49 (1.12) *	0.28 (0.02) *
Use of cholesterol-lowering medication	0.07 (0.03) *	−0.54 (0.29)	−43.59 (0.45) *	0.04 (0.01) *
Medical history of cerebrovascular disease	−0.36 (0.15) *	−0.41 (1.49)	−7.16 (2.31) *	0.01 (0.04)
Medical history of cardiovascular disease	0.21 (0.10) *	1.87 (1.01)	−1.45 (1.58)	0.05 (0.03)
Medical history of renal failure or dialysis	−0.17 (0.18)	−2.07 (1.81)	8.37 (2.82) *	−0.09 (0.05)
Random effect				
Trajectory group × Subject ID [c]				
Standard deviation of intercept	3.34 (0.03)	12.31 (0.11)	24.40 (0.21)	0.35 (0.00)
Standard deviation of residual	0.87 (0.00)	8.99 (0.03)	13.81 (0.04)	0.22 (0.00)

Data are expressed as coefficients (standard error). Adjusted for age at the first health checkup, sex, total number of health checkups during the observation period, being well-rested through sleep, use of antihypertensive medication, insulin injections or glucose-lowering medication, cholesterol-lowering medication, and medical history of cerebrovascular disease, cardiovascular disease, renal failure, or dialysis.

Abbreviations: BMI, body mass index; sBP, systolic blood pressure; HbA1c, hemoglobin A1c

* p-value <0.05.

[a] Number of health checkups incorporates a time element, where “3” corresponds to the third health checkup.

[b] Total number of health checkups ranged from 10 to 14 owing to the 14-year observation period.

[c] We created a variable by nesting the “trajectory group” and “Subject ID” (e.g., “trajectory groups − subject ID”) and used it as a random effect.
