# Supplementary material for: Trajectory Patterns of Three Lifestyle Behaviors and Subsequent Health Conditions in Japanese Adults: A Retrospective Longitudinal Study Using a Health Checkup Database
Source: JMA J. 2024 Oct 3;7(4):506–17. doi: 10.31662/jmaj.2024-0076 (PMC11543322; doi:10.31662/jmaj.2024-0076)
Supplement: Supplementary Figure 5.1, Supplement Figure 5.2, Supplement Table 5.1, Supplement Table 5.2 [file 2433-3298-7-4-0506-s005.pdf]

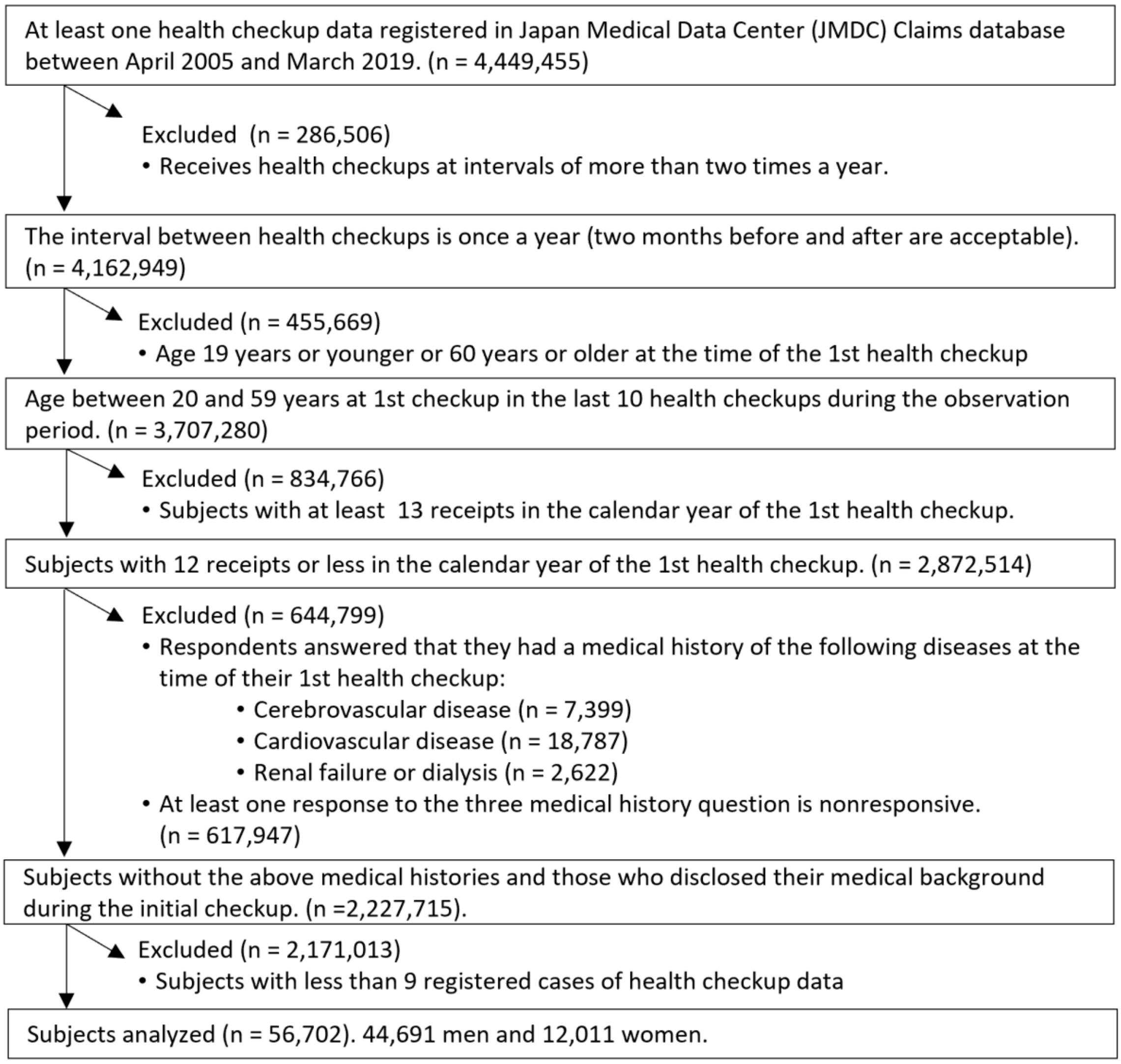

Supplementary Figure 5.1. Flowchart of the study subjects in the sensitivity analysis for inclusion of subjects with one year or more interval between health checkups.

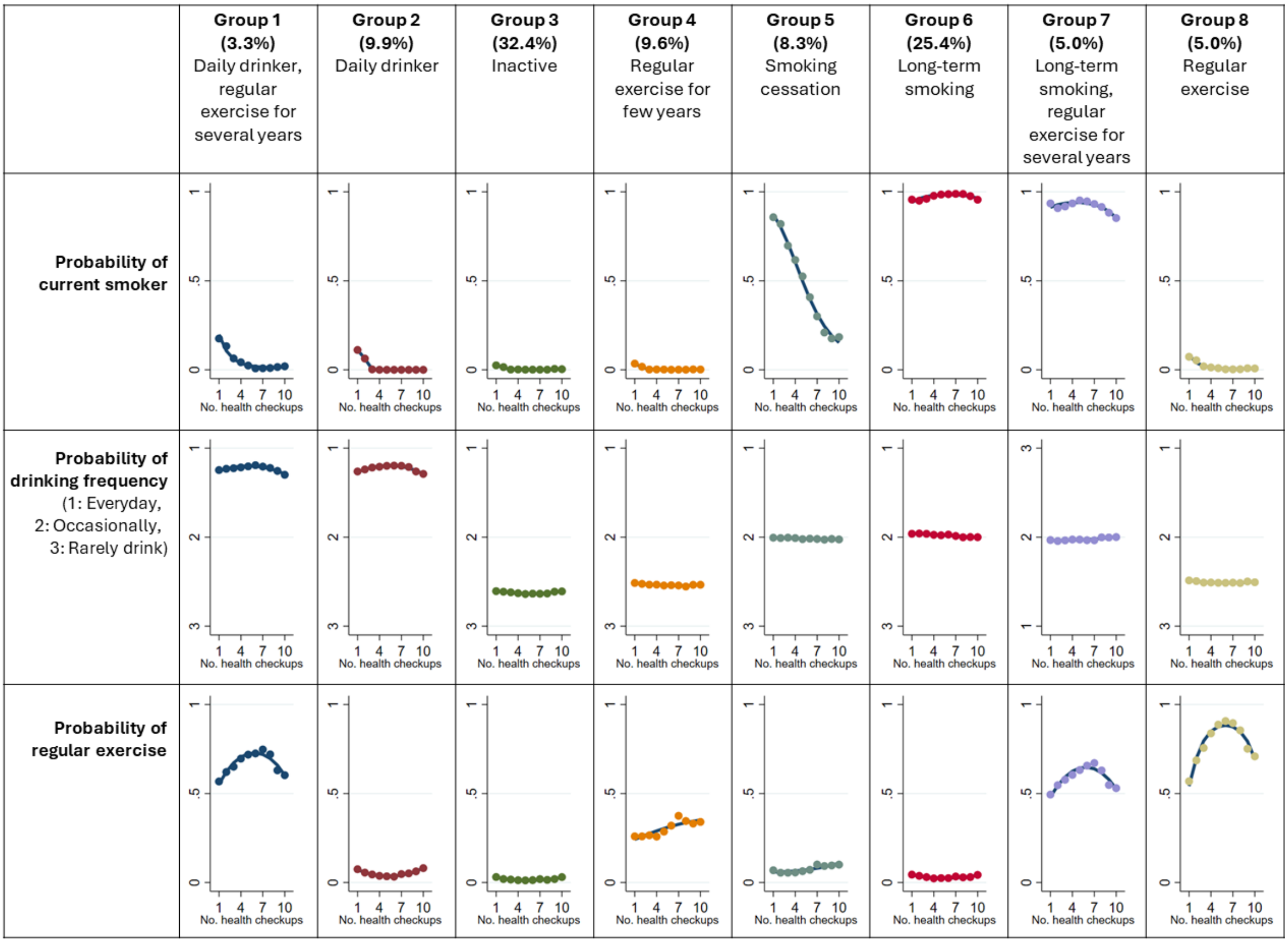

Supplementary Figure 5.2. Multitrajectories of current smoking, frequency of alcohol drinking, and regular exercise in the sensitivity analysis for inclusion of subjects with one year or more interval between health checkups.

Supplementary Table 5.1. Characteristics of health checkup at 1st by group divided by multi-trajectory modeling in the sensitivity analysis for inclusion of subjects with one year or more interval between health checkups.

Variables, %	All (n = 56,702)	Trajectory group								
		Healthy lifestyle (Group 0, n=631)	Daily drinker, regular exercise for several years (Group 1, n=1,888)	Daily drinker (Group 2, n=5,615)	Inactive (Group 3, n=18,362)	Regular exercise for a few years (Group 4, n=5,431)	Smoking cessation (Group 5, n=4,700)	Long-term smoking (Group 6, n=14,416)	Long-term smoking, regular exercise for several years (Group 7, n=2,846)	Regular exercise (Group 8, n=2,813)
Sex, n (%)										
Male	78.8	39.9	91.8	91.4	62.2	64.3	91.9	95.7	94.8	66.2
Female	21.2	60.1	8.2	8.6	37.8	35.7	8.1	4.3	5.2	33.8
Age (years)										
20–29	16.6	6.3	4.9	7.4	19.4	17.4	20.3	17.5	18.0	13.7
30–39	28.8	14.0	19.1	25.0	32.3	27.7	28.6	30.3	24.8	23.0
40–49	42.4	61.7	51.5	50.3	39.6	43.8	38.1	40.8	43.2	47.1
50–59	12.1	18.1	24.5	17.3	8.7	11.2	13.0	11.4	14.1	16.2
Frequency of drinking										
Daily	24.6	0.0	77.2	78.0	2.7	4.9	28.8	34.4	31.0	4.5
Occasional	29.2	36.5	16.4	18.5	32.3	31.7	32.5	27.9	28.5	34.4
None or rarely	40.0	63.6	1.2	1.0	60.8	50.4	31.8	31.8	29.2	48.5
Missing	6.3	0.0	5.3	2.6	4.2	13.0	6.8	5.9	11.3	12.7
Current smoker										
No	60.7	100.0	82.4	88.4	97.6	96.6	14.4	4.4	6.7	92.4
Yes	39.3	0.0	17.6	11.6	2.4	3.4	85.6	95.6	93.3	7.5
Missing	0.0	0.0	0.0	0.1	0.0	0.0	0.0	0.0	0.0	0.0
Exercise at least 2 days a week for at										

least 30 min										
No	76.0	0.0	40.1	86.2	86.2	60.7	81.2	85.4	43.4	36.7
Yes	12.9	100.0	54.0	7.0	2.4	25.7	6.2	4.0	42.3	49.2
Missing	11.0	0.0	5.9	6.8	11.5	13.6	12.6	10.6	14.3	14.1
Improvement of lifestyle										
I do not intend to improve	15.3	35.2	13.6	10.7	15.1	14.9	12.5	16.5	19.0	18.2
I intend to improve (generally within 6 months)	29.7	14.1	19.5	31.6	33.3	26.1	30.0	31.2	21.8	19.6
I intend to improve in the near future (generally within a month) and have begun to do so gradually	7.3	9.5	6.3	4.8	8.6	10.3	6.1	5.8	7.8	8.0
Already working on improvement (less than 6 months)	4.1	6.0	4.5	2.4	4.2	7.2	3.2	2.7	5.9	7.5
Already working on improvement (more than 6 months)	4.2	17.8	11.2	3.8	2.9	6.2	3.3	1.8	7.5	12.7
Missing	39.4	17.4	45.0	46.7	36.0	35.3	44.9	42.1	38.0	33.9

Data are shown as % in column.

The calendar year of the first checkup differed for each subject. The number of subjects in each calendar year was 157 in 2007, 3669 in 2008, 45494 in 2009, 1733 in 2010, and 11 in 2011.

Supplementary Table 5.2. Linear mixed-effect model estimates for anthropometric and laboratory over the course of 10 health checkups in the sensitivity analysis for inclusion of subjects with one year or more interval between health checkups.

Parameters	BMI	sBP	LDL-cholesterol	HbA1c
Fixed effect				
Intercept	21.95 (0.22) *	107.40 (0.80) *	78.38 (1.83) *	4.87 (0.03) *
Trajectory group				
Group 0: Healthy lifestyle	Reference	Reference	Reference	Reference
Group 1: Daily drinker, regular exercise for several years	0.20 (0.16)	2.25 (0.59) *	0.11 (1.32)	−0.17 (0.02) *
Group 2: Daily drinker	−0.02 (0.14)	2.63 (0.54) *	1.22 (1.20)	−0.15 (0.02) *
Group 3: Inactive	0.15 (0.14)	1.13 (0.52) *	4.34 (1.15) *	−0.04 (0.02)
Group 4: Regular exercise for a few years	0.64 (0.14) *	1.21 (0.54) *	5.19 (1.20) *	−0.02 (0.02)
Group 5: Smoking cessation	0.05 (0.14)	1.02 (0.55)	4.54 (1.22) *	−0.06 (0.02) *
Group 6: Long-term smoking	0.02 (0.14)	1.57 (0.52) *	4.18 (1.16) *	−0.06 (0.02) *
Group 7: Long-term smoking, regular exercise for several years	0.55 (0.15) *	1.35 (0.57) *	3.73 (1.27) *	−0.05 (0.02) *
Group 8: Regular exercise	0.43 (0.15) *	0.82 (0.57)	1.76 (1.26)	−0.05 (0.02) *
No. of health checkups [a]	0.04 (0.00) *	0.44 (0.04) *	1.60 (0.07) *	0.00 (0.00)
Interaction of No. of health checkups × Trajectory group				
Group 0: Healthy lifestyle	Reference	Reference	Reference	Reference
Group 1: Daily drinker, regular exercise for several years	0.01 (0.00) *	0.11 (0.05) *	−0.94 (0.08) *	0.01 (0.00) *
Group 2: Daily drinker	0.04 (0.00) *	0.11 (0.04) *	−0.89 (0.07) *	0.01 (0.00) *
Group 3: Inactive	0.06 (0.00) *	0.00 (0.04)	−0.35 (0.07) *	0.01 (0.00) *
Group 4: Regular exercise for a few years	0.03 (0.00) *	−0.05 (0.04)	−0.39 (0.07) *	0.01 (0.00) *
Group 5: Smoking cessation	0.12 (0.00) *	0.14 (0.04) *	−0.60 (0.08) *	0.01 (0.00) *
Group 6: Long-term smoking	0.06 (0.00) *	0.01 (0.04)	−0.94 (0.07) *	0.01 (0.00) *
Group 7: Long-term smoking, regular exercise for several years	0.03 (0.00) *	0.02 (0.05)	−1.02 (0.08) *	0.01 (0.00) *
Group 8: Regular exercise	0.01 (0.00) *	−0.01 (0.05)	−0.46 (0.08) *	0.00 (0.00) *

Age at 1st health checkup	0.03 (0.00) *	0.31 (0.01) *	1.02 (0.01) *	0.02 (0.00) *
Women sex	−2.04 (0.04) *	−9.69 (0.13) *	−6.98 (0.31) *	−0.11 (0.01) *
Total No. of health checkups during the observation period [b]	−0.04 (0.02) *	0.14 (0.06) *	−0.43 (0.13) *	0.01 (0.00) *
Being well-rested through sleep	0.03 (0.00) *	0.23 (0.04) *	0.46 (0.07) *	−0.01 (0.00) *
Use of antihypertensive medication	0.18 (0.01) *	−4.77 (0.10) *	−5.17 (0.17) *	0.06 (0.00) *
Use of insulin injections or glucose-lowering medication	−0.46 (0.02) *	−0.15 (0.17)	−9.49 (0.29) *	0.24 (0.01) *
Use of cholesterol-lowering medication	0.16 (0.01) *	−0.19 (0.10)	−28.56 (0.17) *	0.06 (0.00) *
Medical history of cerebrovascular disease	−0.18 (0.04) *	−1.59 (0.43) *	−5.16 (0.72) *	−0.04 (0.02) *
Medical history of cardiovascular disease	−0.11 (0.03) *	−1.83 (0.30) *	−12.42 (0.52) *	0.01 (0.01)
Medical history of renal failure or dialysis	−0.05 (0.07)	−1.11 (0.67)	1.83 (1.14)	−0.06 (0.03) *
Random effect				
Trajectory group × Subject ID [c]				
Standard deviation of intercept	3.27 (0.01)	10.87 (0.04)	25.96 (0.09)	0.44 (0.00)
Standard deviation of residual	0.88 (0.00)	9.17 (0.01)	15.28 (0.02)	0.30 (0.00)

Data are expressed as coefficients (standard error). Adjusted for age at the first health checkup, sex, total number of health checkups during the observation period, being well-rested through sleep, use of antihypertensive medication, insulin injections or glucose-lowering medication, cholesterol-lowering medication, and medical history of cerebrovascular disease, cardiovascular disease, renal failure, or dialysis.

Abbreviations: BMI, body mass index; sBP, systolic blood pressure; HbA1c, hemoglobin A1c

* p-value < 0.05.

[a] Number of health checkups incorporates a time element, where “3” corresponds to the third health checkup.

[b] Total number of health checkups ranged from 10 to 14 owing to the 14-year observation period.

[c] We created a variable by nesting the “trajectory group” and “Subject ID” (e.g., “trajectory groups − subject ID”) and used it as a random effect.
